# How the Skin Thickness and Thermal Contact Resistance Influence Thermal Tactile Perception

**DOI:** 10.3390/mi10020087

**Published:** 2019-01-25

**Authors:** Congyan Chen, Shichen Ding

**Affiliations:** School of Automation, Southeast University, Nanjing 210096, China; 220161453@seu.edu.cn

**Keywords:** thermal tactile perception, surface roughness, skin thickness, thermal perception reproduction

## Abstract

A few experimental studies on thermal tactile perception have shown the influence of the thermal contact resistance which relates to contact surface roughness and pressure. In this paper, the theoretical influence of the skin thickness and the thermal contact resistance is studied on the thermal model describing the temperature evolution in skin and materials when they come in contact. The thermal theoretical profile for reproducing a thermal cue for given contact thermal resistance is also presented. Compared to existing models of thermal simulation, the method proposed here has the advantage that the parameters of skin structure and thermal contact resistance in target temperature profiles can be adjusted in thermal perception simulation according to different skin features or surface roughness if necessary. The experimental results of surface roughness recognition were also presented.

## 1. Introduction

Thermal perception is a rich, emotive, and entirely silent information source. For example, when our hands touch objects, thermal perceptions can provide information about their thermal characteristics, and help us recognize materials [1]. More, it could be used as an alternative mobile feedback channel when required, as it is silent for quiet environments, especially in electromagnetic interference case where monitors or headsets cannot work normally.

To simulate exchanging information by thermal tactile, some thermal displays have been developed for the reproduction of the thermal perception when a finger is in contact with a virtual or a remote real object [2,3]. Different thermal properties make different thermal profiles which result in different thermal tactile perceptions [4].

The relationship between the contact temperature evolution and the thermal characteristics have been studied to develop thermal feedback systems [5,6]. The works presented theoretical and experimental study of a model of heat exchange during hand-object interactions, and particularly evaluated by comparing the theoretical values of temperature changes to those experimentally measured [7,8]. The authors also studied how the contact area and contact pressure during hand-object interactions affected the skin temperature changes.

When a finger contacts a material, not only the thermal characteristics of the skin and the material but also the skin physical structure and their contact state have an influence on the heat exchange during hand-object interactions. There are some significant factors affecting the heat exchange.

The work [9] proposed a model for heat transfer occurring between the finger skin and any given surface based on an electrical analogy, and discussed the comparison between the model and some experimental results by considering various phenomena like the applied pressure by the finger, the speed of the blood circulation, the interface state. The experimental results [10] have shown that there was a small change in skin temperature as a function of the surface roughness of the contact material. Some analyses of the relation between the skin temperature change and contact pressure in a thermal display have been also presented with the aid of an infrared thermal measurement system [11]. A quantified model for local and overall thermal sensations in non-uniform thermal environments is also proposed in [12].

However, some features still should be specified. For example, what influence do the finger skin physical characteristics (The thickness of skin, the surface roughness) exert on thermal perception during hand-object interactions?

The thermoreceptors which function as thermal sensors are scattered between the dermis and epidermis [13]. The thermal perception originates from the temperature drop and its change rate at thermoreceptors, which relates to physical and thermal properties, initial temperature difference, thermal contact resistance, and other factors [14].

Finite-element calculations have been applied to simulation in the case of thermal contact resistance in order to simulate flat and smooth surfaces of objects with different properties in virtual reality [2]. The fingertip surface roughness was measured and the thermal contact resistance of the finger was estimated based on an infrared camera during interaction phases to study the influence of surface properties on thermal tactile perception [15]. With the addition of thermal contact resistance to the thermal model, the temperature profiles of the skin and materials become more realistic. 

However, the role of a fingertip skin thickness and the roughness of contact surface, which influences the temperature drop at thermoreceptors, should be investigated more. The authors in [13] have considered the thickness of the epidermis and dermis to model the temperature responses at the cold receptors. The thermal model was studied from the consideration that the skin is not regarded as homogeneous but as consisting of three layers of tissue that differ in terms of heat flux: epidermis, dermis, and endodermis [16]. 

The skin thickness and the roughness of contact surface were both considered in the present paper in order to study the thermal responses of the fingertip as it makes contact with materials with different surface roughness. We believe it is helpful in modelling thermal tactile perception with considering these factors.

## 2. Thermal Modelling 

**Figure 1:**
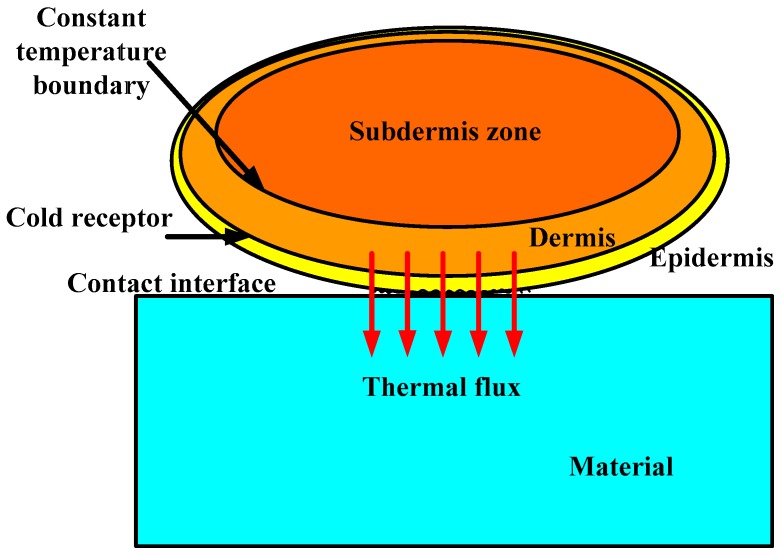
The condition of contact between a fingertip and a material.

The condition of contact between a fingertip and a material is shown in Figure 1. The fingertip has three layers: the inner layer is well known as the subdermal zone; the middle layer is the dermis; and the outer layer is the epidermis. The thermal contact resistance is specifically considered here.

As the subdermal zone is with large heat capacity and low heat conductivity, its temperature will remain stable normally when the outside thermal stimulus changes. A thermoreceptor is a sensory receptor that helps us get “cold” or “hot” sensations. The temperature and its change rate at warm or cold thermoreceptors are the main source of cold or hot sensation [17].

Cold thermoreceptors are much more numerous than warm thermoreceptors by a ratio of up to 30:1, and respond to the decreases in temperature over a temperature range of 5–43 °C [18,19]. The different depths of cold thermoreceptor may bring about different temperature drops. However, the actual depth is dependent on the certain thickness of skin.

The thermal exchange between the skin and a material in contact with is a transient process and is dominated by heat conduction. Normally heat flows from the skin to materials. And the thermal contact resistance between the fingertip and materials is involved unavoidably in real contact. So, it should be also considered in thermal modelling.

These total thermal resistances result in less temperature drop at thermoreceptors than that in theory. For above considerations, an experimental system has been constructed to measure the surface roughness [8]. And a device was also developed to implement the skin-object thermal contact resistance measurement [20].

**Figure 2:**
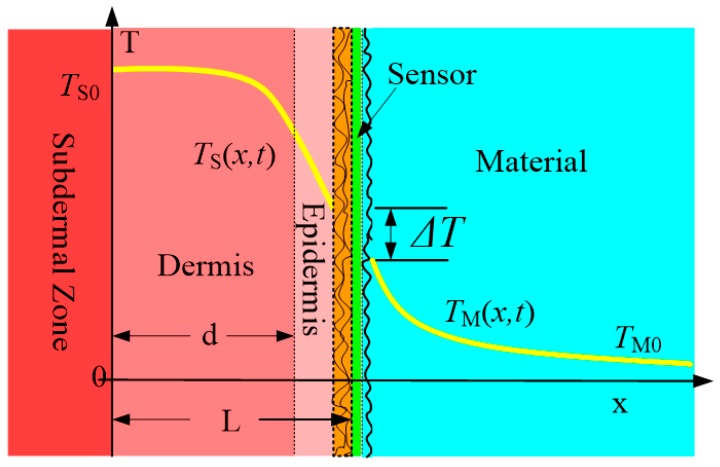
Thermal contact system.

The whole thermal contact system with thermal resistance considered can be modelled as shown in Figure 2. 

$T_{S}\left(x,t\right)$ and $T_{M}\left(x,t\right)$ refer to finger temperature and material temperature, and $T_{S0}$ and $T_{M0}$ are their initial values. Here L is the thickness of skin, and d is the thickness of its dermis. And there is a thermal sensor between the finger and material to measure the interface temperature. Here, the thermal resistance which results in some temperature drop ΔT is denoted as R here.

Let $\lambda_{i}$ be the thermal conductivity, $c_{i}$ be the specific heat, and $\rho_{i}$ be the density, then $\alpha_{i}=\frac{\lambda_{i}}{\rho_{i}c_{i}}$ is known as the thermal diffusion coefficient, and $\beta_{i}={\left(\lambda_{i}\rho_{i}c_{i}\right)}^{\frac{1}{2}}$ is the thermal contact coefficient, here i=S and M represents skin and material respectively. So, the governing Equations of the skin and material can be expressed as:

(1a) $$\frac{\partial \theta_{S}(x,t)}{\partial t}=\alpha_{S}\frac{\partial^{2}\theta_{S}(x,t)}{\partial x^{2}},\;\left\{\begin{array}{c} \theta_{S}\left(x,t\right)=1,t=0\\ \theta_{S}\left(x,t\right)=1,x=0 \end{array}\right\}$$

(1b) $$\frac{\partial \theta_{M}\left(x,t\right)}{\partial t}=\alpha_{M}\frac{\partial^{2}\theta_{M}\left(x,t\right)}{\partial x^{2}},\;\left\{\begin{array}{l} \theta_{M}\left(x,t\right)=0,t=0\\ \theta_{M}\left(x,t\right)=0,x\to \infty \end{array}\right\}$$

where the relative residual temperature is defined:

$$\theta_{i}\left(x,t\right)=\frac{T_{i}\left(x,t\right)-T_{M0}}{T_{S0}-T_{M0}},\;i=S,M.$$

When t>0, the boundary conditions are $\theta_{S}\left(L^{-},t\right)=\theta_{M}\left(L^{+},t\right)+\Delta \theta$ at the contact interface, and $-\lambda_{S}\frac{\partial \theta_{S}\left(x,t\right)}{\partial x}|x=L^{-}=-\lambda_{M}\frac{\partial \theta_{M}\left(x,t\right)}{\partial x}|x=L^{+}=\frac{\theta_{S}\left(L^{-},t\right)-\theta_{M}\left(L^{+},t\right)}{R}$, t>0. By introducing Laplace transforms to Equations (1a) and (1b), they are transformed to the differential Equations in variable x:

(2a) $$\frac{d^{2}{\overset{ˉ}{\theta }}_{S}\left(x,s\right)}{dx^{2}}=\frac{1}{\alpha_{S}}\left[s{\overset{ˉ}{\theta }}_{S}\left(x,s\right)-1\right]$$

(2b) $$\frac{d^{2}{\overset{ˉ}{\theta }}_{M}\left(x,s\right)}{dx^{2}}=\frac{1}{\alpha_{M}}s{\overset{ˉ}{\theta }}_{M}\left(x,s\right)$$

where s is the common Laplace complex variable.

Let $\mu =\sqrt{\frac{\alpha_{S}}{\alpha_{M}}}$, $\beta =\frac{\lambda_{S}}{\lambda_{M}\mu }=\frac{\beta_{S}}{\beta_{M}}$, $H_{1}=\frac{\beta +1}{R\lambda_{S}}$, $H_{2}=\frac{\beta -1}{R\lambda_{S}}$, $\gamma =\frac{\beta -1}{\beta +1}=\frac{H_{2}}{H_{1}}$ and take the inverse Laplace transform of Equations (2a) and (2b). The approximate solution of the temperature in skin and material can be gotten as (3a) and (3b) according to the Laplace transform table in [21].

(3a) $$\theta_{S}\left(x,t\right)=1-\frac{\beta_{M}}{\beta_{S}+\beta_{M}}\overset{\infty }{\underset{n=0}{\sum }}{\left(-1\right)}^{n}\gamma^{n}\left\{erfc\left[\frac{\left(2n+1\right)L-x}{2\sqrt{\alpha_{S}t}}\right]-erfc\left[\frac{\left(2n+1\right)L+x}{2\sqrt{\alpha_{S}t}}\right]-e_{SR}\right\}$$

(3b) $$\theta_{M}\left(x,t\right)=\frac{\beta_{S}}{\beta_{S}+\beta_{M}}\overset{\infty }{\underset{n=0}{\sum }}{\left(-1\right)}^{n}\gamma^{n}\left\{erfc\left[\frac{2nL-\mu \left(x-L\right)}{2\sqrt{\alpha_{S}t}}\right]+erfc\left[\frac{\left(2n+2\right)L+\mu \left(x-L\right)}{2\sqrt{\alpha_{S}t}}\right]-e_{MR}\right\}$$

where $e_{SR}=e^{H_{1}\left(L-x\right)+H_{1}{}^{2}\alpha_{S}t}erfc\left[H_{1}\sqrt{\alpha_{S}t}+\frac{L-x}{2\sqrt{\alpha_{S}t}}\right]-e^{H_{1}\left(L+x\right)+H_{1}{}^{2}\alpha_{S}t}erfc\left[H_{1}\sqrt{\alpha_{S}t}+\frac{L+x}{2\sqrt{\alpha_{S}t}}\right]$,

$e_{MR}=e^{H_{1}\mu \left(x-L\right)+H_{1}{}^{2}\alpha_{S}t}erfc\left[H_{1}\sqrt{\alpha_{S}t}+\frac{\mu \left(x-L\right)}{2\sqrt{\alpha_{S}t}}\right]-e^{H_{1}\left[2L+\mu \left(x-L\right)\right]+H_{1}{}^{2}\alpha_{S}t}erfc\left[H_{1}\sqrt{\alpha_{S}t}+\frac{2L+\mu \left(x-L\right)}{2\sqrt{\alpha_{S}t}}\right]$.

The temperatures in the fingertip skin and the material can be written as respectively:

(4a) $$T_{S}\left(x,t\right)=T_{S0}-\left(T_{S0}-T_{M0}\right)\frac{1-\gamma }{2}\overset{\infty }{\underset{n=0}{\sum }}{\left(-1\right)}^{n}\gamma^{n}\left\{erfc\left[\frac{\left(2n+1\right)L-x}{2\sqrt{\alpha_{S}t}}\right]-erfc\left[\frac{\left(2n+1\right)L+x}{2\sqrt{\alpha_{S}t}}\right]-e_{SR}\right\}$$

(4b) $$T_{M}\left(x,t\right)=T_{M0}+\left(T_{S0}-T_{M0}\right)\frac{1+\gamma }{2}\overset{\infty }{\underset{n=0}{\sum }}{\left(-1\right)}^{n}\gamma^{n}\left\{erfc\left[\frac{2nL-\mu \left(x-L\right)}{2\sqrt{\alpha_{S}t}}\right]+erfc\left[\frac{\left(2n+1\right)L+\mu \left(x-L\right)}{2\sqrt{\alpha_{S}t}}\right]-e_{MR}\right\}.$$

It is evident that the temperature drop at the thermoreceptors is dependent on not only $T_{S0}$, $T_{M0}$, $\beta_{S}$, $\beta_{M}$, $\alpha_{S}$, $\alpha_{M}$, but also L, d, and the thermal resistance R. Here $\beta_{S}$ and $\alpha_{S}$ are the thermal parameters characterizing the skin of fingertip, L and d are the physical parameters characterizing the skin of fingertip, and R is the thermal resistance parameter characterizing the contact state for thermal conductivity. 

Before simulating using the above thermal model, the thermal contact resistance can be given from the following relation [15]:

(5) $$R_{c}=\frac{0.8R_{q}}{\lambda_{SF}\Delta a}{\left(\frac{H}{P}\right)}^{0.95}$$

where $\lambda_{SF}=\frac{2\lambda_{S}\cdot \lambda_{a}}{\lambda_{S}+\lambda_{a}}$ (W/m∙k) is the harmonic mean thermal conductivity of the contact interface, $R_{q}={\left[R_{S}^{2}+R_{a}^{2}\right]}^{0.5}$ is the effective root mean square surface roughness, Δa is the effective absolute average surface asperity slope, $H=12.5\;g/{\mathrm{mm}}^{2}$ is the skin microhardness, and P is the contact pressure. Here some parameters were adopted directly in the following simulation from the reference [15]: Δa=0.3, $H=12.5\;g/{\mathrm{mm}}^{2}$, $R_{S}=21.69\;\mu m$, and $R_{a}$ is for the surface roughness of material. The contact pressure is given with a contact force of 2 N and contact area of 135 mm^2^. 

**Figure 3:**
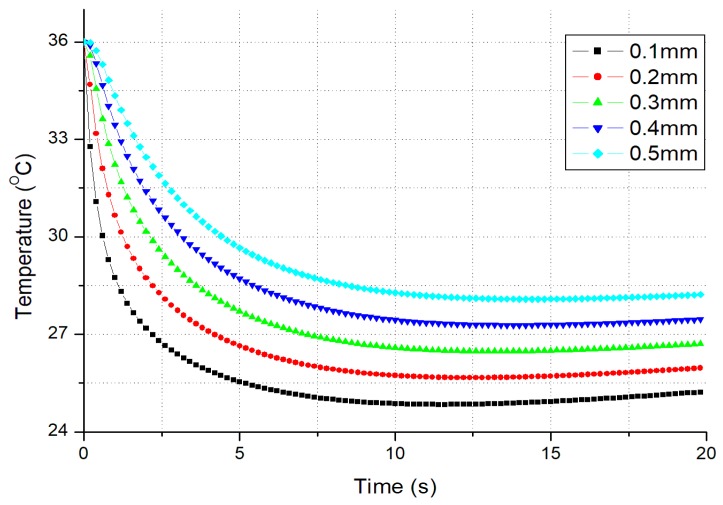
The temperature evolutions at thermoreceptors for different thicknesses of epidermis (Material = SS with absolutely smooth surface). SS—stainless steel.

The theoretical temperature evolutions of stainless steel (SS) for different thicknesses of epidermis are shown in Figure 3. As denoted above, L is the thickness of fingertip skin, and d is the thickness of dermis, then L−d is the thickness of epidermis. Here the initial temperatures of finger and material are set as 36 °C and 20 °C, respectively. The thermal characteristics applied in the simulation have been listed in Table 1. The simulation results show that the temperature evolutions at thermoreceptors are quite different for different thicknesses of epidermis.

The terms $e_{SR}$ and $e_{MR}$ are factors for thermal contact resistance. Comparing with the model without considering thermal contact resistance, the factor $e_{SR}$ will bring a temperature drop loss at thermoreceptors:

(6) $$\left|\Delta T_{S}\left(x,t\right)\right|=\frac{\beta_{M}}{\beta_{S}+\beta_{M}}e_{SR}\left|T_{S0}-T_{M0}\right|.$$

For the same material, the temperature drop at thermoreceptors depends on not only the initial temperature difference between skin and the material but also their thermal contact state. The temperature drop will reduce with the increase of the thermal contact resistance.

**Figure 4:**
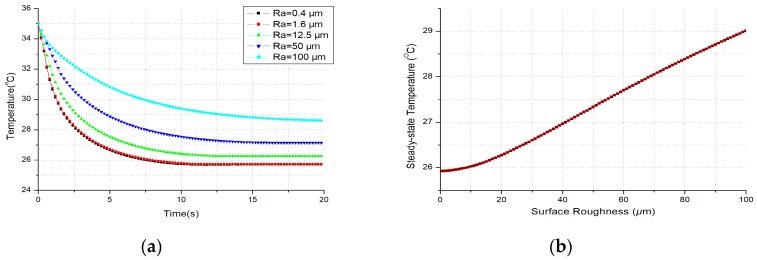
The simulations for different surface roughness of SS: (**a**) The temperature evolutions at thermoreceptors; (**b**) The relationship between surface roughness and steady-state temperature at thermoreceptors.

The theoretical temperature evolutions at thermoreceptors with different surface roughness of stainless steel (SS) are illustrated in Figure 4a, where each temperature drop and its change rate reduce obviously with the increase of the surface roughness. And in Figure 4b, the steady-state temperature (replaced with the value at 20th second in the simulation) at thermoreceptors becomes higher with the increase of surface roughness for the heat flux conducted out of the skin becomes less.

It is shown in Figure 4b that the different surface roughness results in different steady-state temperatures at thermoreceptors. Namely the absolute temperature drops for the same material are also different after first several seconds of contact. And this may bring some different degrees of thermal tactile perception.

**Remark** **1.** *From Figure 3, besides the initial temperature difference $\left|T_{S0}-T_{M0}\right|$ between skin and material and their thermal properties, one factor influencing temperature evolution at thermoreceptors is the thickness of skin. The thicker the skin, the smaller is the temperature drop at thermoreceptors.*

Skin thickness varies considerably according to the race, age, sex and region of the body surface. For example, for Korean population, the thickness of epidermis varies from 31 μm to 637 μm, while the thickness of dermis varies from 469 μm to 1942 μm [22]. So, the factor of skin thickness should be studied in thermal simulation.

**Remark** **2.** *Another factor is the thermal contact resistance. It is obvious in Figure 4a that each temperature evolution at thermoreceptors is quite different from others as the contact surface roughness of each material sample is not the same. So, does each steady-state temperature drop. Both of the theory and simulation results show that the temperature drop at the thermoreceptors becomes less with the increase of the thermal contact resistance. So, the influence of thermal contact resistance should be also considered in modelling for thermal tactile perception.*

Now consider how to simulate the thermal perception of a given material with the initial temperature $\;T_{M0}\;$ when considering the influence of both skin thickness and thermal contact resistance. 

To reproduce an appointed thermal cue via a thermal tactile display, the contact temperature should be controlled to track the corresponding target temperature profile. A thermal sensor was situated between the fingertip skin and the material to measure the interface temperature. The theoretical profile of the interface temperature can be gotten from the following Equation:

(7) $$T_{M}\left(x,t\right)|L^{+}\leftarrow x=T_{M0}+\left(T_{S0}-T_{M0}\right)\frac{1+\gamma }{2}\left\{erfc\left[\frac{\mu \left(x-L\right)}{2\sqrt{\alpha_{S}t}}\right]+erfc\left[\frac{2L+\mu \left(x-L\right)}{2\sqrt{\alpha_{S}t}}\right]-e_{MR}\right\}|L^{+}\leftarrow x.$$

**Figure 5:**
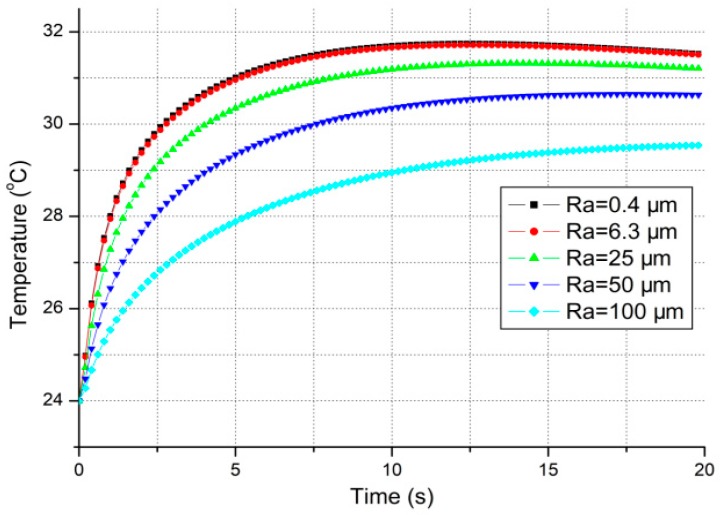
The theoretical interface temperature profiles for different surface roughness (material = SS, $R_{s}=21.69\;\mu m$).

Equation (7) derives from Equation (4b) when x approaches $L^{+}$. $T_{M}\left(x,t\right)|L^{+}\leftarrow x$ denotes the theoretical interface temperature of material side. It should be noted that the thermal contact resistance can be adjusted in thermal perception simulation. The theoretical interface temperature profiles for different surface roughness of SS are illustrated in Figure 5. 

With the increase of surface roughness, the thermal contact resistance increases and the heat flux out of the skin becomes less, and the steady-state temperature drop becomes less. So, the surface roughness of material also results in some difference in thermal tactile perception. 

From the definition in [4], the theoretical relative recognizing profiles with thermal resistance at thermoreceptors can be gotten as:

(8) $$\psi \left(t\right)=\frac{T_{S0}-T_{S}\left(d,t\right)}{T_{S0}-T_{M0}}=\frac{1+\gamma }{2}\left\{erfc\left[\frac{L-d}{2\sqrt{\alpha_{S}t}}\right]-erfc\left[\frac{L+d}{2\sqrt{\alpha_{S}t}}\right]-e_{SR}\right\}$$

**Figure 6:**
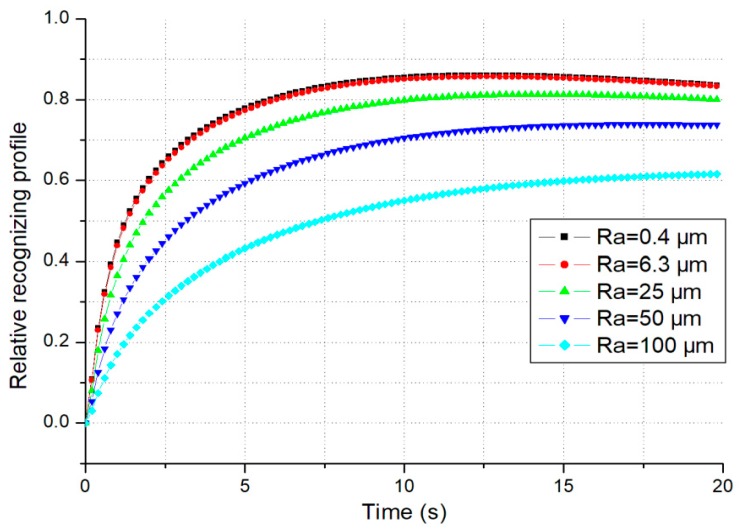
The relative thermal recognizing profiles (material = SS, $R_{s}=21.69\;\mu m$).

In ideal case, the relative recognizing profiles with the same thermal characteristics are consistent for different initial temperatures. However, in real case, the thermal contact resistance has some influence on relative recognizing profile as discussed above. The relative recognizing profiles of SS with different surface roughness are illustrated in Figure 6.

**Remark** **3.** *It is feasible to apply relative thermal recognizing profiles to thermal tactile perception simulation with adjusting the parameter of thermal contact resistance. Due to inter-individual variations, it is difficult to set the initial skin temperatures exactly or thermal contact resistances to specific ones. However, we can manage to measure them and provide some approximate values. As soon as they are determined, the theoretical profiles are then gotten, and the corresponding thermal cues can be reproduced by a thermal tactile display.*

## 3. Experiments

The two experiments have been presented here. The first experiment is designed to study the influence of the thermal contact resistance on the thermal recognizing profile. The second one is aimed to verify the influence of the skin thickness and investigate the difference between real and simulated surface roughness recognition.

### 3.1. Influence of the Surface Roughness on the Thermal Recognizing Profiles

In order to evaluate the influence of thermal contact resistance on thermal modelling, an experiment was designed to investigate the evolution of interface temperature for different surface roughness of the same material. One set of temperature profile was measured for different surface roughness of SS. 

**Figure 7:**
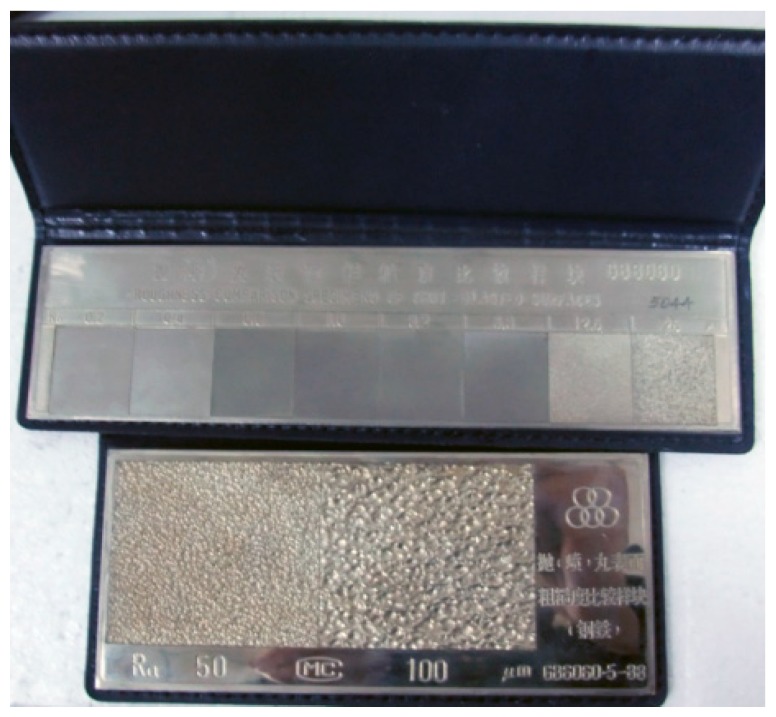
The samples with different surface roughness (material = SS).

The object with different surface roughness was standard samples processed by surface shot-peening, whose surface sizes are 20 mm × 23 mm or 50 mm × 46 mm with the thickness of 3 mm. As is shown in Figure 7, it is divided into 8 parts of different surfaces roughness. The object is made of nickel alloy using precision electroforming technology and has an advantage of high hardness, good abrasion resistance and good rust prevention. It makes this experiment safe and reliable.

The participant cleaned his right hand before starting the experiment. A platinum thin-film thermal sensor (polyimide with foil backing, Minco S651) was glued to the fingertip of the right index finger with a biocompatible cyanoacrylate to decrease thermal resistance of contact. 

**Figure 8:**
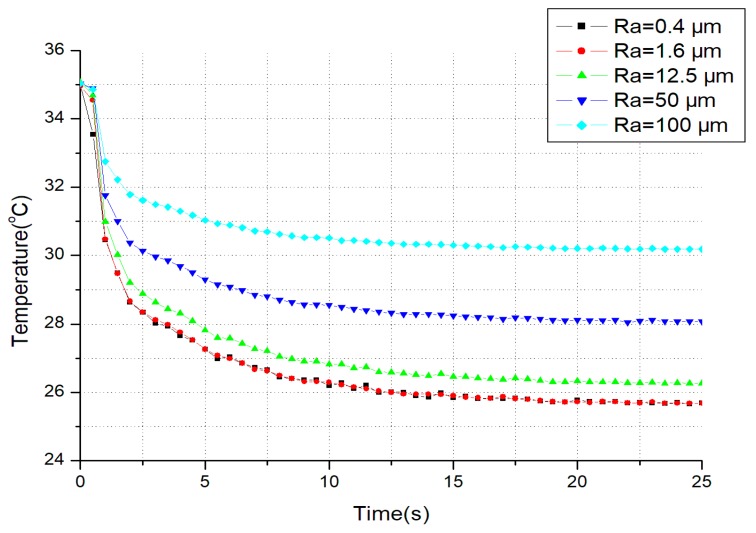
The experimental temperature evolution for different surface roughness (material = SS).

The initial temperature of skin was about 35 °C, warmed and maintained with a hot-water bag beforehand. The room temperature was maintained at 24 °C, and also measured by a thermal sensor whenever necessary. The participant was instructed to place his right index fingertip on each sample in turn. The contact time for each trial lasted more than 25 s. However, the change of the contact temperature was only recorded in first 25 s for the transient process is over. Every temperature evolution was measured by the sensor, and illustrated in Figure 8. 

The experimental temperature evolutions in Figure 8 are similar to those in Figure 4. With the increase of the surface roughness of SS, the steady-state temperature drop (approximately replaced by the values at 25th second) also becomes less. This verifies that the surface roughness of SS affects the thermal tactile perception during hand-object interactions. The perception will become weaker because of the increase of the thermal resistance. 

The comparisons between simulation and experimental data at 20 s are shown in Table 2. There are slight differences between simulation and experiment. Due to the limited sensor size and the spherical surface of the roughness samples (50 μm and 100 μm), the actual contact pattern including contact area and the contact pressure is different from others by degrees, resulting in a large deviation from the theoretical value. 

### 3.2. Experiment of Recognition of Different Surface Roughness

The second experiment was designed to measure subject’s ability to identify SS samples with different surface roughness, and investigate the difference between real and simulated surface roughness recognition. The participants are thirty normal healthy adults including twelve women and eighteen men aged between 18 and 45 years in experiments. They were all right-handed, but with different occupations, for example, student, teacher, worker, farmer, etc. Before the experiment, they were simply trained to contact samples with about 2 N pressure expertly. Besides, their skin thickness was estimated by calculating the dimension of the skin’s bio-speckles [23].

According to their skin thickness, the participants are split two groups: group A and group B. Each group has fifteen participants. The group A has five men and ten women, whose skin thickness is smaller than 800 μm, 600–750 μm. The group B has twelve men and three women, whose skin thickness is greater than 800 μm, about 900–1200 μm. 

The experimental material is SS, whose surface roughness is listed in Table 3. 

Each participant’s index finger was first cleaned in order to not interfere with the contact. The participants’ initial fingertip skin temperatures ranged from 35.5 °C to 36 °C, warmed beforehand with a hot-water bag, and measured by a radiation thermometer just before touching. The room temperature was maintained at about 20 °C. In experiment there was a Platinum thin-film thermal sensor (also S651) glued with thermally conductive silicone which can decrease the thermal contact resistance between the sensor and samples. The sensor also helps to prohibit the surface texture tactile perception.

At the beginning, four stainless steel samples with different surface roughness were shown and presented to thirty participants by thermal feedback to become familiar with them.

In the real surface roughness recognition experiment, four stainless steel samples with different roughness were presented to participants randomly with three repetitions of each sample. Participants were forbidden to watch the procedure. When making contact with a sample they were instructed to judge it. No feedback was given regarding the correctness of each judgment. The contact time for each trial was not more than 20 s. After finishing the real material recognition experiment, participants could touch the samples again and reviewed their thermal cues. The results were labeled into two group A and B with real material. 

In the next simulated surface roughness recognition experiment, the theoretical thermal cues are reproduced from the Equation (7). Except the skin thickness, the physical properties of skin and contact state in thermal reproduction were chosen as the same in the above simulation for simplicity. The mean skin thickness was set as 650 μm for group A and 1000 μm for group B, respectively. 

The thermal tactile display device applied to simulate the different thermal cues has been developed [24]. It consists of a Peltier pad, two thermal sensors for ambient temperature and contact temperature, a radiation thermometer for finger temperature and four pressure sensors for the force of touch. The maximum difference of temperature between the cold and hot sides is more than 80 °C. 

The target temperature profiles were given by the theoretical temperature based on Equation (7). As long as the initial skin and material temperatures also their thermal characteristics were set respectively, then the corresponding target temperature profile was specified for the given surface roughness and skin thickness. 

Four samples with different simulated roughness were presented to participants randomly with three trials. After hearing a sound cue, a participant put the right index finger on the sensor attached on the Peltier pad. At the same time, the temperature profile was set to the corresponding target one. The participants took their hands back to the hot-water bag after speaking out the choice of the simulated roughness. The contact time for each trial was also not more than 20 s. 

**Figure 9:**
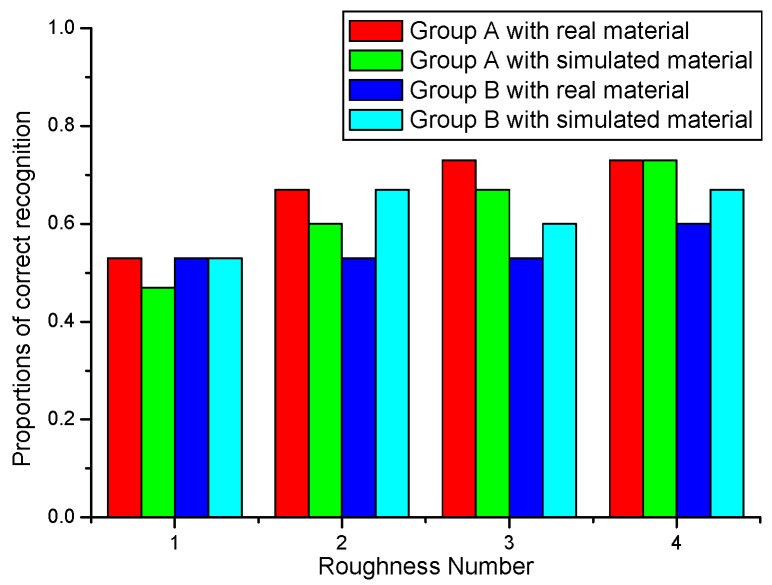
The proportions of correct recognition of two groups for real and simulated SS samples.

The two groups’ responses in term of the correct percentage of roughness recognition for both real and their corresponding simulated samples are illustrated in Figure 9. 

As shown in Figure 9, the correct proportion of surface roughness recognition goes up slowly with the difference of the thermal recognition profiles which become more obvious, as shown in Figure 8. However, the correct proportions of surface roughness recognition are still not quite high. The sample with surface roughness 1# (*R_a_* = 1.6 μm) is often confused with other samples, both for the real and simulated samples. And the results also indicate that when only thermal cues are available, surface roughness recognition is quite not definite even when there is obvious difference of roughness. 

On the other hand, comparing the correct recognition proportions of group A and group B for the same real surface roughness, the proportion of the group A is higher than that of the group B to a certain extent. This means that the skin thickness has some influence on thermal perception. 

As pointed out above, thicker skin will bring about less temperature drop at thermoreceptors in contact with real material. For simulated material, however, this difference becomes less because the temperature drop loss is compensated according to our thermal model.

With the addition of thermal contact resistance to the thermal model, the temperature profiles of the skin and materials become more realistic. Although the different surface roughness can be recognized by the thermal tactile perception to a certain extent, the thermal perception is a complex process. It also relates to the participant’s psychological response. However, the results show at least that the influence of surface roughness on thermal perception is existed basically in experiment. 

Comparing to previous thermal simulation methods, the one proposed here has an advantage that the parameters of skin structure or surface roughness in target temperature profile can be adjusted in thermal simulation experiment according to different skin feature or material surface roughness if necessary. 

## 4. Conclusions

This paper investigated the factors influencing thermal tactile perception based on thermoreceptors. Thickness of skin and thermal contact resistance have been considered in thermal modelling, and the theoretical temperature profiles in skin and material have been presented when they contact with each other. Also, a method has been proposed to reproduce thermal cues for different skin thickness and surface roughness. With the consideration of thermal contact resistance in thermal modelling, the temperature profiles of the skin and materials become more realistic. The experimental results of roughness recognition for real and simulated materials indicate that material roughness and the skin thickness influence the thermal perception. 

## Figures and Tables

**Table 1:** Thermal properties of stainless steel (SS) and skin.

Material	λ (W/(m∙K))	ρ (kg/m^3^)	c (J/(kg∙K))	β (W∙s^1/2^/(m^2^∙K))
SS	14.9	7900	447	7253.71
Skin	0.34	1200	3340	1167.36

**Table 2:** Comparisons between simulation and experimental data.

Roughness (μm)	Simulation Data (°C)	Experiment Data (°C)	Errors (%)
0.4	25.704	25.776	0.280
1.6	25.729	25.751	0.086
12.5	26.269	26.340	0.270
50	27.338	28.116	2.84
100	28.820	30.021	4.17

**Table 3:** Roughness of experimental sample.

Roughness Number	Roughness Value (*R_a_*, μm)
1#	1.6
2#	12.5
3#	50
4#	100
